# Evaluation of the QRS score for diagnosing coronary artery disease in women: A Finnish cardiovascular study

**DOI:** 10.1111/anec.12968

**Published:** 2022-05-17

**Authors:** Serkalem D. Beyene, Kjell C. Nikus, Terho J. Lehtimäki, Mika A. P. Kähönen, Jari J. Viik

**Affiliations:** ^1^ Faculty of Medicine and Health Technology Tampere University Tampere Finland; ^2^ Heart Center Tampere University Hospital Tampere Finland; ^3^ Department of Clinical Physiology Tampere University Hospital, and Faculty of Medicine and Health Technology, Tampere University Tampere Finland

**Keywords:** electrocardiography, exercise test, female, ROC curve

## Abstract

**Background:**

Exercise electrocardiography is a widely used diagnostic modality for diagnosing coronary artery disease. This method has been used for both sexes; however, its diagnostic accuracy in women is limited.

**Methods:**

The study analyzed 332 women participating in the Finnish Cardiovascular Study. Among 332 women, 125 with angiographically proven coronary artery disease (mean age 62.1 ± 9.5 years), 91 with a low likelihood of coronary artery disease (mean age 47.3 ± 13.5 years), and 116 without angiographically proven coronary artery disease (mean age 56.3 ± 9.9 years) were analyzed. The Q, R, S, and ST‐segment changes and QRS score were determined by subtracting the Q, R, S, and ST‐segment amplitudes immediately after the maximal exercise changes from their rest values (Δ). Receiver operating characteristic curve analysis was performed to evaluate the overall diagnostic performance of the parameters for predicting coronary artery disease.

**Results:**

The areas under the receiver operating characteristic curve between coronary artery disease and low likelihood of coronary artery disease groups for the QRS score and ΔSTV5, ΔQaVF, and ΔRaVF were 0.75, 0.73, 0.71, and 0.71, respectively. These areas were lower (0.62, 0.57, 0.60, and 0.60, respectively) between the groups with and without angiographically proven coronary artery disease. QRS score demonstrated the highest sensitivity at 80% specificity (61.5%) and the highest specificity at 80% sensitivity (57.6%).

**Conclusions:**

This study suggests that the QRS and ST‐segment depression have a moderate diagnostic ability to predict coronary artery disease in women. Q and R waves in lead aVF showed good diagnostic ability.

## Graphical abstract

Researchers evaluated the ability of exercise induced QRS score, its composition (Q, R, and S) wave changes and ST‐segment depression in diagnosing coronary artery disease (CAD) in women. They noted that QRS score and ST‐segment depression have a moderate ability to help diagnose CAD, followed by Q and R waves in lead aVF.

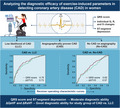

## 1 INTRODUCTION

Recent guidelines recommend exercise electrocardiography (ECG) for the assessment of exercise tolerance, symptoms (angina pectoris, shortness of breath, fatigue or fainting, nausea, burning sensation in the chest, restlessness, or a sense of impending doom), arrhythmias, blood pressure (BP) response, and event risk in selected patients (Knuuti et al., 2020) Compared with popular coronary artery imaging modalities, exercise ECG is widely accessible, less resource‐intensive, cheaper, and does not expose patients or personnel to radiation. Therefore, in clinical practice, exercise ECGs are still widely used for diagnostic purposes in coronary artery disease (CAD). Studies have indicated that ECG can be effective for patients capable of performing a maximal level of exercise without significant ST‐segment changes in the resting ECG (Shaw et al., 2011). This method has been used in both men and women to detect CAD; however, its diagnostic accuracy in women is limited compared with that in men (Svart et al., 2010). This difference can be attributed to the lower exercise capacity (average 2 min less) of women and the lower probability of achieving the target heart rate than men in the same age group. More often, women have pre‐existing resting ST‐segment changes and lower QRS voltage. Additionally, estrogen can affect ST‐segment changes and have a digoxin‐like effect during exercise (Higgins & Higgins, 2007). Women with moderate‐sized breasts may experience excessive ECG motion artifacts from the breasts pushing and pulling on the adjacent skin and precordial ECG leads, which can lead to difficulty in interpreting ST‐segment changes (Higgins & Higgins, 2007). Women have a smaller coronary vessel diameter, which may reduce the maximal flow heterogeneity and potentially decrease ST‐segment changes (Higgins & Higgins, 2007).

Exercise‐induced ST‐segment depression is the most commonly used ECG marker for myocardial ischemia. However, it is less reliable for detecting CAD in women because of its low sensitivity and specificity (Morise & Diamond 1995). A meta‐analysis by Kwok et al. (1999) revealed that exercise ECG was less accurate in women than in men. Based on 19 studies, wherein at least 50 women were subjected to both the exercise test and coronary angiography, the weighted mean sensitivity and specificity were 61% and 70%, respectively. This meta‐analysis also considered 10 studies of exercise ECG, in which both men and women were included. The sensitivity and specificity were 70% and 77%, respectively, and significant difference was observed. This result was in line with that of a previous meta‐analysis by Gianrossi et al. (1989), which included 147 studies involving 24,074 participants, most of whom were men. Here, the sensitivity and specificity were 68% and 77%, respectively.

Exercise‐induced changes in the Q, R, and S waves have been evaluated to improve the efficacy of exercise testing to diagnose CAD. However, researchers have challenged the reliability of amplitude changes in these waves with exercise as a marker of CAD (O'Hara et al., 1984; Wagner et al., 1979; Glazier et al., 1987). Michaelides et al. (1990) tested and proved the hypothesis that considering the composite expression of exercise‐induced changes in Q, R, and S waves might result in higher sensitivity and specificity. This initial report was subsequently supported by other studies (Alvi et al., 2016; Michaelides et al., 2004; Michaelides et al., 2007; Michaelides et al., 2010; Toth et al., 2001; Van Campen et al., 1996); however, most of the population included in these studies were men, and women were under‐represented. This indicates that further studies should be conducted to investigate the diagnostic ability of the QRS score in women.

Therefore, the objective of this study was to evaluate the diagnostic ability of exercise‐induced QRS score and its composition (Q, R, and S) wave changes with conventional ST‐segment depression in Finnish women.

## 2 METHODS

### 2.1 Study cohort

This study analyzed the Finnish Cardiovascular Study data cohort (Nieminen et al., 2006), which was designed and conducted to explore the impact of exercise ECG test markers on the anticipation of events and deaths caused by cardiovascular disease. The database comprised 4178 (2537 men and 1641 women) patients who underwent an exercise test at Tampere University Hospital (TAUH), Tampere, Finland, between October 2001 and December 2008. A total of 332 women (mean age, 56.1 ± 12.6 years) participated in the study. Based on angiography‐proven results and clinical history, the patients were divided into three groups:

#### 2.1.1 Angiographically proven CAD (CAD)

This group initially comprised 215 female patients who were selected from patients who had previously undergone angiography and had ≥50% luminal diameter narrowing in at least one major epicardial coronary artery. To the best of our knowledge, the time period for significant changes in the status of the coronary arteries has not been defined. Therefore, we chose a cutoff of 180 days arbitrarily and then excluded 73 women who had a time difference of more than 180 days between the exercise ECG and angiography. Seventeen patients were excluded from the study because we were unable to determine the amplitude values of the Q or S waves in these patients. Ultimately, 125 women were included in this group.

#### 2.1.2 No CAD on angiography (no CAD)

The criterion for inclusion was no main stenosis of the coronary arteries. In addition, the time difference between the exercise ECG and angiography was not greater than 180 days. A total of 116 women were included in this group.

#### 2.1.3 Low likelihood of CAD (LLC)

Patients who had no previous cardiac events, who did not use any cardiac medications (β‐blockers, calcium channel blockers, angiotensin‐converting enzyme inhibitors or angiotensin 2 antagonists, short‐ or long‐acting nitrates, diuretics, and digitalis), who did not show chest pain during exercise, and with low probability of CAD after the exercise test based on the opinion of a supervised physician. In total, 91 women were included in this study group.

The database comprised 4178 (2537 men and 1641 women) patients who underwent an exercise test at Tampere University Hospital (TAUH), Tampere, Finland, between October 2001 and December 2008. A total of 332 women (mean age, 56.1 ± 12.6) participated in the study. Based on angiography‐proven results and clinical history, the patients were divided into three groups:

#### 2.1.4 Angiographically proven CAD (CAD)

This group initially comprised of 215 female patients who were selected from patients who had previously undergone angiography and had ≥50% luminal diameter narrowing in at least one major epicardial coronary artery. To the best of our knowledge, the time period for significant changes in the status of the coronary arteries has not been defined. Therefore, we chose a cutoff of 180 days arbitrarily and then excluded 73 women whose time difference between the exercise ECG and angiography was greater than 180 days. Seventeen patients were excluded from the study because we were unable to determine the amplitude values of the Q or S waves in these patients. Ultimately, 125 women were included in this group.

#### 2.1.5 No CAD on angiography (no CAD)

The criterion for inclusion was no main stenosis of the coronary arteries. In addition, the time difference between the exercise ECG and angiography was not greater than 180 days. A total of 116 women were included in this group.

#### 2.1.6 Low likelihood of CAD (LLC)

Patients who had no previous cardiac events, who did not use any cardiac medications (β‐blockers, calcium channel blockers, angiotensin‐converting enzyme inhibitors or angiotensin 2 antagonists, short‐ or long‐acting nitrates, diuretics, and digitalis), who did not show chest pain during exercise, and with low probability of CAD after the exercise test based on the opinion of a supervised physician. In total, 91 women were included in this study group.

### 2.2 Exercise test protocol

The routine exercise ECG stress test at the TAUH was performed on a bicycle ergometer with electrical brakes, wherein the Mason–Likar modification of the standard 12‐lead system was applied. The entire exercise test covered the resting phase, wherein the patient was laid in the supine position for 10 min until the recovery phase, which was started immediately after the exercise phase and lasted at least 5 min after the exercise. The ECG exercise test was performed while the patients were on a bicycle ergometer with electrical brakes. Initially, the workload varied from 20 to 30 W and increased every minute by 10–30 W. The exercise tests were sign‐ and symptom‐limited maximal tests using the recommended criteria for termination (Nieminen et al., 2006; Salokari et al., 2019). A high‐resolution ECG at 500 Hz was continuously recorded with the CardioSoft exercise ECG system (Version 4.14, GE Healthcare,) during the entire exercise test. The results of the exercise ECG were analyzed using modified CASE (GE Healthcare).

### 2.3 ECG variables measurement

Individual depolarization changes in Q, R, and S waves, composite QRS score, and ST‐segment amplitude were analyzed in this study. The amplitudes of Q, R, and S waves and ST‐segment were determined using CASE (GE Healthcare) before the exercise phase and immediately after the exercise in leads aVF and V5. There were a significant number of NAN (not a number) values of the Q and S amplitudes in both leads. To impute these values, row data were plotted using data recorded before and immediately after the exercise. The average amplitude value was then obtained visually from the plotted trend. Q, R, and S waves and ST‐segment amplitude values immediately after exercise were subtracted from those immediately before the exercise phase. The differences were termed delta Q (ΔQ), delta R (ΔR), delta S (ΔS), and delta ST‐segment (ΔST). ΔQ and ΔS were then subtracted from ΔR in both aVF and V5 leads. Finally, the QRS score was calculated by summing the values for leads aVF and V5. The formula for QRS score (mV) is as follows: (ΔR – ΔQ – ΔS) aVF + (ΔR – ΔQ – ΔS) V5 (Michaelides et al., 1990).

### 2.4 Statistical methods

Continuous study variables are expressed as means and standard deviations. The Chi‐square test was performed for the comparison of discrete variables of the exercise test and the analysis of significant differences among different groups in the clinical characteristics of the study data. Quantitative variables were examined using an independent sample t‐test; if the distribution was not normal, the Mann–Whitney U test was used. To evaluate the diagnostic ability of the QRS score and its composition, Q‐, R‐, and S‐wave changes with that of conventional ST‐segment deviation, receiver operating characteristic (ROC) curves were used, and the values of the area under the curves were calculated. Paired‐sample area differences under the ROC curves were determined between the QRS score and the remaining ECG variables involved in both study groups to analyze the significant differences between them. The sensitivity values at 80% specificity and specificity values at 80% sensitivity were also determined for each variable. All tests were considered significant at the level of *α* = 0.05. Statistical analysis was performed using IBM SPSS Statistics (IBM Corp. Released 2019. IBM SPSS Statistics for Windows, Version 26.0. Armonk, NY: IBM Corp).

## 3 RESULTS

### 3.1 Baseline clinical characteristics of the study population

Table 1 presents the baseline clinical characteristics and statistical comparisons between the study groups, where the no‐CAD and LLC groups were compared with the CAD group. Age, body mass index (BMI), maximum heart rate (HRmax), and maximum (max) workload are expressed as mean ± standard deviation, whereas other parameters are presented as percentages of patients in the specific group. The patients in the CAD category were older, more often on a β‐blocker or a calcium (Ca) channel blocker, had a history of acute myocardial infarction, and reached a maximum heart rate faster than those in the no‐CAD and LLC categories (*p* < .001). Comparing patients in the LLC and CAD study groups, of the patients in the LLC category, only one patient was diabetic. The LLC patients had lower BMI, did not use any cardiac medications, did not demonstrate chest pain during exercise, and had maximum exercise performance of the two groups (*p* < .001). Patients in the no‐CAD category had a lower rate of ACE inhibitor use and demonstrated better exercise performance than those in the CAD group.

**TABLE 1:** Mean, standard deviation, percentages, and p‐values of clinical characteristics of the study groups

Characteristics	CAD (*n* = 125)	No‐CAD (*n* = 116)	*p*‐values	LLC (*n* = 91)	*p*‐values
Age (years)	62.1 ± 9.5	56.3 ± 9.9	<.001	47.3 ± 13.5	<.001
BMI (kg/m^2^)	28.3 ± 5.2	27.2 ± 4.5	.089	26.0 ± 5.0	.001
No smoking habit	82.3	90.4	.110	80.0	.680
Diabetes, type 2	13.2	8.9	.297	1.1	<.001
History of previous MI	37.9	0	<.001	0	<.001
HR max (beats/min)	135.5 ± 27.6	154.3 ± 21.7	<.001	167.5 ± 16.9	<.001
ACE inhibitors	27.4	15.8	.029	0	<.001
Max workload (W)	85.2 ± 36.7	98.5 ± 34.1	.005	124.4 ± 41.6	<.001
ATR antagonists	15.3	9.6	.269	0	<.001
Diuretics	28.2	14.9	.012	0	<.001
Ca channel blockers	22.6	7.9	.001	0	<.001
β‐blockers	84.7	50.9	<.001	0	<.001
HRT	16.2	22.8	.356	13.3	.272
Chest pain in exercise test	34.1	23.7	.345	0	<.001

The first set of *p‐values* is obtained by comparing parameters between the CAD and no‐CAD groups, whereas the second set is obtained by comparing parameters between the CAD and LLC groups.

Abbreviations: ACE, angiotensin‐converting enzyme; ATR, angiotensin II receptor; BMI, body mass index; CAD, coronary artery disease; HRT, hormone replacement therapy; HR, heart rate; LLC, low likelihood of CAD; MI, myocardial infarction; n, number of patients.

### 3.2 ECG variables

Table 2 presents the mean values, standard deviations, and p‐values for QRS score; Q‐, R‐, and S‐wave changes; conventional ST‐segment depression; and comparisons between the CAD and the other two groups. *p*‐value for the QRS score, ΔQaVF, ΔRaVF, ΔRV5, ΔSaVF, and ΔSTV5 was <.001, which indicated a good performance of CAD detection in comparison between the CAD and LLC study groups. Comparison between CAD and angiographically proven no‐CAD groups indicated no significant difference in the ECG variables ΔSaVF, ΔSTaVF, and ΔSTV5.

**TABLE 2:** Statistical comparisons of exercise ECG test parameters between CAD and other groups

ECG parameters (mV)	CAD (mean ± SD)	No‐CAD (mean ± SD)	*p*‐values	LLC (mean ± SD)	*p*‐values
ΔQaVF	−0.00 ± 0.11	0.03 ± 0.10	.007	0.04 ± 0.07	<.001
ΔQV5	0.0 ± 0.06	0.02 ± 0.10	.028	0.04 ± 0.10	.044
ΔRaVF	0.02 ± 0.18	0.08 ± 0.17	.007	0.17 ± 0.22	<.001
ΔRV5	−0.02 ± 0.30	0.03 ± 0.22	.032	0.15 ± 0.26	<.001
ΔSaVF	0.07 ± 0.13	0.09 ± 0.09	.147	0.12 ± 0.18	<.001
ΔSV5	0.16 ± 0.16	0.19 ± 0.17	.034	0.22 ± 0.15	.002
ΔSTaVF	−0.04 ± 0.05	−0.04 ± 0.07	.337	−0.01 ± 0.09	.001
ΔSTV5	−0.06 ± 0.08	−0.05 ± 0.08	.054	0.00 ± 0.12	<.001
QRS score	0.25 ± 0.55	0.45 ± 0.52	.002	0.74 ± 0.61	<.001

The first set of *p‐values* is obtained by comparing ECG parameters between the CAD and no‐CAD groups while the second set is obtained by comparing ECG parameters between the CAD and LLC groups.

Abbreviations: CAD, coronary artery disease; LLC, low likelihood of CAD. The LLC and non‐CAD groups were compared with the CAD group.

**FIGURE 1:**
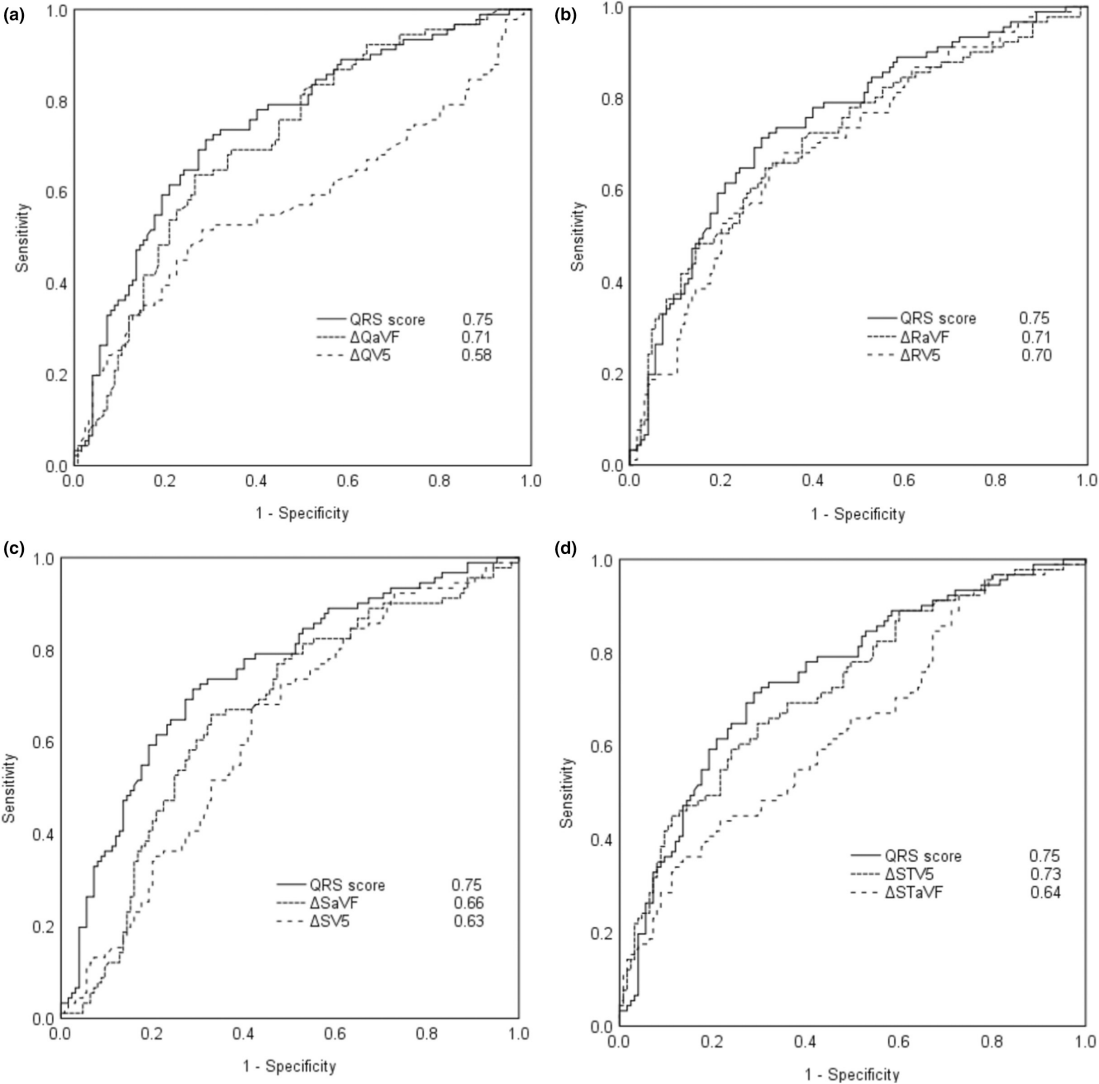
Receiver operating characteristic (ROC) curves and areas under the curves in comparison between (a) QRS score versus Q wave in lead aVF and V5, (b) QRS score versus R wave in lead aVF and V5, (c) QRS score versus S wave in lead aVF and V5, and (d) QRS score versus ST‐segment depression in lead aVF and V5 considering study groups of CAD versus LLC

**FIGURE 2:**
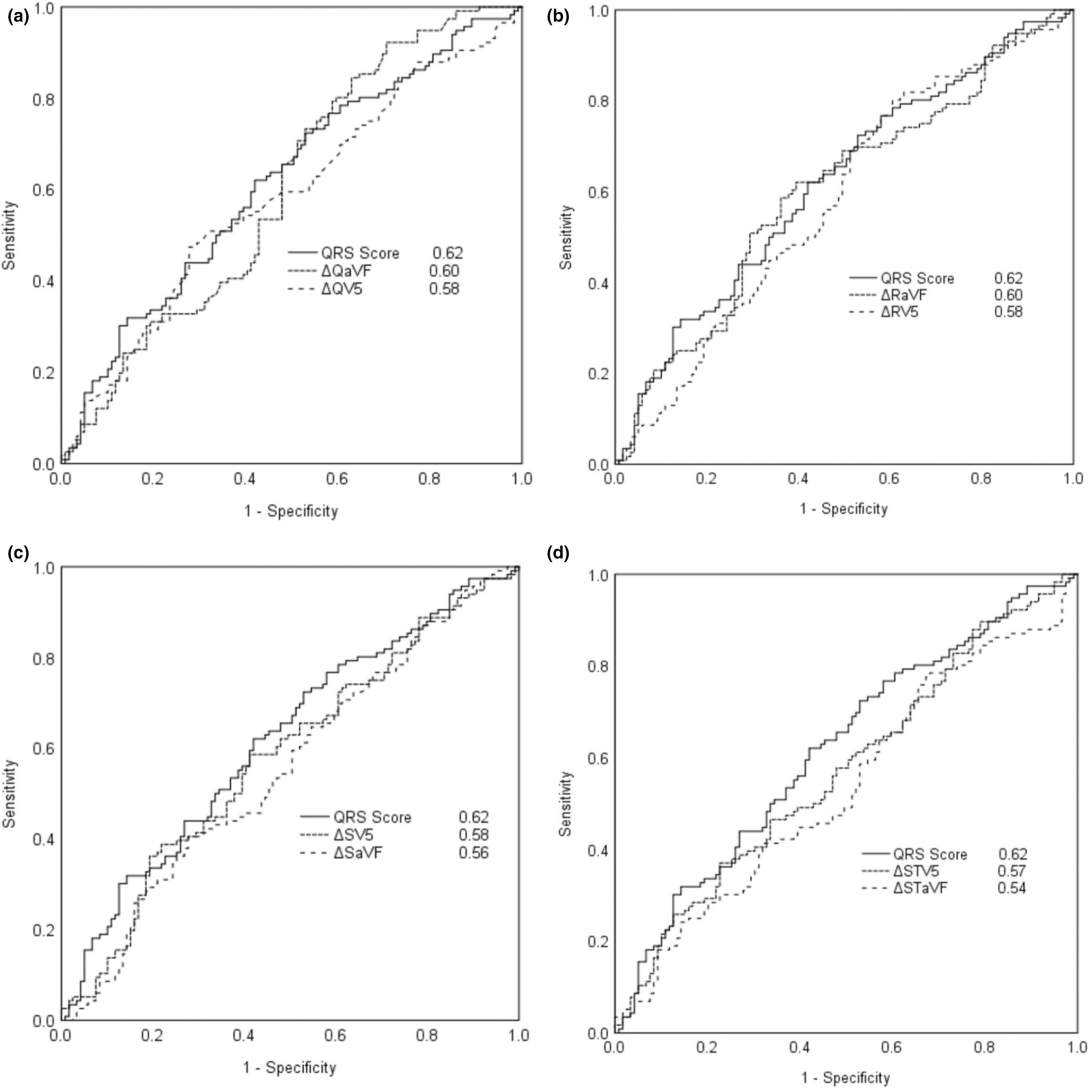
Receiver operating characteristic (ROC) curves and areas under the curves in comparison between (a) QRS score versus Q wave in lead aVF and V5, (b) QRS score versus R wave in lead aVF and V5, (c) QRS score versus S wave in lead aVF and V5, and (d) QRS score versus ST‐segment depression in lead aVF and V5 in considering study groups of CAD versus angiographically proven no‐CAD

The area under the ROC curves for each individual ECG variable in leads aVF and V5 and for the QRS score using CAD and LLC groups are presented in Figure 1. The ROC areas for the QRS score, ΔQaVF, ΔRaVF, and ΔSTV5 were 0.75, 0.71, 0.71, and 0.73, respectively. The results indicated a good performance capacity of these variables in CAD detection. Between the CAD and the angiographically proven no‐CAD groups, the area under the ROC curves for the ECG variables in leads aVF and V5 are presented in Figure 2. The QRS score demonstrated the highest value (0.62), but most variables had nearly the same area under the ROC curves.

Table 3 presents the sensitivity at 80% specificity and specificity at 80% sensitivity for each ECG variable. The QRS score revealed the highest sensitivity at 80% specificity 61.5% and the highest specificity at 80% sensitivity 57.6% for the CAD and LLC groups.

**TABLE 3:** Sensitivity values at 80% specificity and specificity values at 80% sensitivity for the exercise ECG variables

ECG parameters	CAD vs. LLC		CAD vs. no‐CAD	
	Specificity at 80% sensitivity (%)	Sensitivity at 80% specificity (%)	Specificity at 80% sensitivity (%)	Sensitivity at 80% specificity (%)
ΔQaVF	50.4	48.4	40.3	31.0
ΔQV5	19.2	41.8	27.7	29.3
ΔRaVF	46.4	50.5	22.7	27.6
ΔRV5	42.4	46.2	38.7	26.7
ΔSaVF	47.2	43.0	24.4	29.3
ΔSV5	40.0	34.1	27.7	36.2
ΔSTaVF	32.8	40.7	26.1	28.4
ΔSTV5	45.6	50.0	26.9	29.3
QRS score	57.6	61.5	35.3	33.6

Abbreviations: CAD, coronary artery disease; LLC, low likelihood of CAD.

Table 4 reveals the paired‐sample area difference under the ROC curves between QRS score and other ECG variables involved in the study. The p‐value between QRS scores and the ECG variables ΔQaVF, ΔRaVF, and ΔSTV5 indicated that there were no significant differences among them. Significant differences were noticed in QRS score and the ECG variables ΔQV5, ΔRV5, ΔSaVF, ΔSV5, and ΔSTaVF between the CAD and LLC groups. Regarding the CAD and no‐CAD groups, no significant differences were observed between QRS scores and all studied ECG variables.

**TABLE 4:** Paired‐sample area difference under the ROC curves

		CAD vs. LLC		CAD vs. no‐CAD	
ECG parameters		ROC areas	*p*‐values	ROC area	*p*‐values
QRS score –	ΔQaVF	0.71	.427	0.60	.736
	ΔQV5	0.58	.001	0.58	.474
(CAD vs. LLC 0.75	ΔRaVF	0.71	.137	0.60	.578
CAD vs. no‐CAD 0.62)	ΔRV5	0.70	.032	0.58	.123
	ΔSaVF	0.66	.026	0.56	.079
	ΔSV5	0.63	.002	0.58	.273
	ΔSTaVF	0.64	.050	0.54	.180
	ΔSTV5	0.73	.661	0.57	.438

The first set of ROC area and *p‐values* is obtained from the comparison between QRS score and the listed ECG variables for the CAD and LLC groups. The second set is obtained from the comparison between QRS score and the listed ECG variables for the CAD and no‐CAD groups.

## 4 DISCUSSION

The results of this study reveal that exercise‐induced amplitude changes in the QRS score and ST‐segment depression demonstrated moderate diagnostic ability for CAD in the female population compared with its composition (Q, R, and S) wave changes.

Previous studies have revealed that exercise‐induced QRS scores improve the limited diagnostic ability of exercise tests (Alvi et al., 2016; Michaelides et al., 1990; Michaelides et al., 2004; Michaelides et al., 2010; Toth et al., 2001; Van Campen et al., 1996). Michaelides et al. (1990) introduced a new CAD index based on exercise‐induced QRS changes. This study revealed that exercise‐induced changes in Q‐, R‐, and S‐wave amplitudes and the ST‐segment depression have lower sensitivities of 75%, 65%, 70%, and 62%, respectively, and lower specificities of 50%, 55%, 10%, and 70%, respectively. In a previous study, exercise‐induced changes in the QRS complex revealed 75% sensitivity in patients who were analyzed retrospectively and 86% sensitivity in patients analyzed prospectively. In addition, 73% and 79% specificities were observed for patients analyzed retrospectively and prospectively, respectively. This result is supported in a study by van Campen et al. (1996), who observed sensitivity and specificity of abnormal QRS score and ST‐segment depression of 88% and 55%, respectively, and 84% and 83%, respectively, for the detection of CAD. Other researchers have also revealed that the QRS score improves the limited diagnostic and prognostic ability of exercise testing and its extent in relation to myocardial ischemia (Michaelides et al., 2004; Michaelides et al., 2010; Toth et al., 2001). According to these studies, the QRS score significantly increased the limited diagnostic capacity of conventional ST‐segment depression and individual depolarization changes in Q, R, and S waves. However, most participants in these studies were men. A clinical study with a small number of women and the potential effects of workup bias by Michaelides et al. (2007) revealed that the QRS score can improve the limited diagnostic ability of treadmill exercise testing in women. However, to the best of our knowledge, this is the only study involving the women population, which suggests that further evaluation is needed to determine whether the QRS score can improve the limited diagnostic ability of CAD in the female population. Our study involved 332 women who were categorized into three groups based on the angiography‐proven result and clinical history: angiographically proven CAD, no CAD with angiography, and LLC. Exercise‐induced QRS scores were compared not only with conventional ST‐segment depression but also with individual depolarization changes in Q, R, and S waves in both aVF and V5 leads. The results of this study indicate that the QRS score and ST‐segment depression provide moderate CAD diagnosis ability in the female population. The values of the ROC areas suggest that ΔQaVF and ΔRaVF provide good CAD detection ability than the rest individual depolarization changes in Q, R, and S waves for the CAD and LLC study groups. The area under the ROC curves between the angiographically proven no‐CAD and CAD study groups, QRS score, and ΔSTV5 revealed a limited diagnostic ability for CAD. Sensitivity at 80% specificity and specificity at 80% sensitivity of the QRS score were observed to be better than the rest ECG variables between the CAD and LLC groups.

Our findings are in line with those of a previous report (Michaelides et al., 2007). However, the QRS score revealed only moderate CAD detection ability in our population. In addition, the patients involved in this study underwent the bicycle ergometer exercise test mode. Studies have demonstrated that the two modes of exercise test (treadmill and bicycle) have significant differences in the diagnostic ability in different populations (Abiodun et al., 2015; Balogun et al., 1997; Basset & Boulay, 2000; Maeder et al., 2005; Myers et al., 1991; Wicks et al., 1978). This study indicates that the QRS score and ST‐segment depression have moderate diagnostic ability for CAD in women; however, these results require further evaluation in the future considering the degree of severity of CAD (number of obstructed coronary arteries).

The primary limitation of this study is that 83.9% of the angiographically proven CAD patients used β‐blocker medication, and 53.2% of these patients continued its use during the test. These medications may affect the amplitude changes in ECG variables.

In conclusion, the results of this study reveal that the QRS score and conventional ST‐segment depression have a moderate ability to help diagnose CAD in women. Q and R waves in lead aVF also demonstrated good CAD detection ability in the study sample between the CAD and LLC groups compared with the other individual depolarization changes.

## 6 CONFLICT OF INTEREST

No conflict of interest/none declared.

## 7 AUTHOR CONTRIBUTION

Made substantial contribution to conception and design, analysis and interpretation of the data. Involved in drafting and writing the manuscript. Given final approval of the version to be published. Agreed to be accountable for all aspects of the work in insuring that questions related to the accuracy or integrity of the work are appropriately investigated and resolved.

8

## 9 ETHICAL APPROVAL

The study protocol was approved by the Ethics Committee of the Hospital District of Pirkanmaa, Finland. Patients who participated both in exercise tests and in the study provided written informed consent prior to the interview and measurements, as stated in the Declaration of Helsinki. A detailed description of the study has been published by Nieminen et al. (2006).

## Data Availability

Data available on request due to privacy/ethical restrictions. The data that support the findings of this study are available on request from the corresponding author. The data are not publicly available due to privacy or ethical restrictions.
